# hpci improves reproducibility of computational biology research by integrating Continuous Integration with High Performance Computing clusters

**DOI:** 10.1093/bioinformatics/btag720

**Published:** 2026-10-09

**Authors:** Ignatius Jeppe Menzies, Eric Urng, George Bouras, Robert Moss, John Reeves, Leonard D Goldstein

**Affiliations:** Data Science Platform, Garvan Institute of Medical Research, Darlinghurst, New South Wales 2010, Australia; Data Science Platform, Garvan Institute of Medical Research, Darlinghurst, New South Wales 2010, Australia; School of Medicine, College of Health, Adelaide University, Adelaide, South Australia 5011, Australia; Department of Surgery—Otolaryngology Head and Neck Surgery, Central Adelaide Local Health Network, Adelaide, South Australia 5070, Australia; Infectious Disease Dynamics Unit, Melbourne School of Population and Global Health, The University of Melbourne, Parkville, Victoria 3010, Australia; Swarbrick Lab, Cancer Ecosystems Program, Garvan Institute of Medical Research, Darlinghurst, New South Wales 2010, Australia; Data Science Platform, Garvan Institute of Medical Research, Darlinghurst, New South Wales 2010, Australia; St Vincent’s Clinical School, Faculty of Medicine, University of New South Wales, Darlinghurst, New South Wales 2010, Australia

## Abstract

**Motivation:**

The reproducibility crisis in bioinformatics and computational biology can be mitigated by using Continuous Integration (CI), a proven software engineering practice, to automate the testing of analysis pipelines and models. However, the adoption of CI in bioinformatics is limited as existing solutions are typically tightly linked to specific CI platforms, specific HPC job schedulers, or require actions from HPC administrators.

**Results:**

We developed hpci, a platform-agnostic, open-source utility that integrates CI platforms (e.g. GitHub Actions, GitLab CI) with common HPC job schedulers (Slurm, PBS). hpci operates as a standard unprivileged user via SSH, and requires no cluster-side installation. It terminates on the CI runner using the exact same exit code generated by the HPC job, providing feedback to researchers and preventing downstream CI steps if there are errors. We demonstrate hpci’s utility across five diverse computational biology workflows on HPC, highlighting the value of automated testing and continuous analysis for verifying reproducibility in bioinformatics workflows.

**Availability:**

hpci is implemented in Haskell and is available under the GPL-3.0 licence. Source code and binaries can be downloaded from https://github.com/Garvan-Data-Science-Platform/hpci.

Source code is archived in Software Heritage via Zenodo (https://zenodo.org/records/19587650).

## 1 Introduction

It is a widely held view that there is a reproducibility crisis in science, and especially within biomedical research (Baker 2016, Perkel 2020, Cobey *et al*. 2024). This lack of reproducibility presents a barrier to the translation of research to clinical treatments that improve healthcare (Dirnagl *et al*. 2022). A major driver of this crisis is the highly iterative and non-linear nature of the research process. While developing a research paper it is common to re-run parts of the analysis with additional data, adjusted parameters, or different analysis methods. As the analysis pipeline evolves, irreproducibility can be introduced by human error; inaccurate tracking of which outputs were derived from which version of the analysis code, changes in underlying software, and insufficient testing of data management and analysis code changes (Sandve *et al*. 2013, Trišović *et al*. 2022).

These challenges are not unique to scientific research; in response to similar challenges software engineers developed a process called Continuous Integration (CI) to automate software compilation and testing while developing software (Shahin *et al*. 2017). On each ‘push’ to a remote code repository, the compilation and test processes are run from scratch in a remote server typically with a ‘fresh’ software environment. This requires that compilation and test processes are automated (i.e. no manual steps), that the code is tracked with source version control, and that all software dependencies and environment variables are specified in code. CI provides automated verification that code changes do not introduce errors and, due to source version control, maintains an accurate record of the code, compilation configuration, and output artefacts as the software evolves. While traditionally applied to software development, CI methodologies can be repurposed to verify scientific computational pipelines, ensuring that data processing, analysis, visualization and reporting steps can be run from end-to-end on a remote server, thus verifying that the research is reproducible (sometimes called “Continuous Analysis”) (Beaulieu-Jones and Greene 2017, Rule *et al*. 2019, Kim *et al*. 2022, Braga *et al.* 2023, de Visser *et al*. 2023, Moreau *et al.* 2023, Malladi *et al*. 2024). However, there can be challenges to adopting CI in some research disciplines. Due to the vast increase in data availability of the past 20 years, the computationally intensive nature of many bioinformatics analysis algorithms, and the recent integration with deep learning and foundation models (including large language models), the majority of computational biology data analysis requires the use of high performance computing (HPC) platforms (Du *et al*. 2006, Yin *et al*. 2017, Castrigano *et al*. 2020, Wei *et al*. 2026). Unfortunately integrating cloud-native CI platforms with secure isolated HPC environments is challenging for software applications let alone for Continuous Analysis workflows (Sharif *et al*. 2020, Gamblin and Katz 2022, Nuyujukian 2023, Caspart *et al.* 2026).

While solutions exist to bridge this gap, current approaches typically have properties that are likely to limit widespread adoption by computational biologists. Tools are often tightly coupled to specific CI systems and/or specific job schedulers (Moutsatsos *et al*. 2017, Sampedro *et al*. 2018, Sharif *et al*. 2020, Pachev *et al*. 2022, Marcus 2024, Hayot-Sasson *et al*. 2025; Exascale Computing Project—Jacamar CI https://gitlab.com/ecp-ci/jacamar-ci). Furthermore, other approaches require HPC administrators to open REST APIs, install privileged authorisation software directly on the clusters, or apply a shared identity infrastructure across the CI system and the HPC environment (Adamson *et al*. 2024; Exascale Computing Project—Jacamar CI https://gitlab.com/ecp-ci/jacamar-ci; Slurm Workload Manager REST API Reference 2024 https://slurm.schedmd.com/rest_api.html); such requests are routinely denied due to strict facility security constraints and administrative friction (Gamblin and Katz 2022).

To address this critical infrastructure gap, we developed hpci: a platform-agnostic open-source software tool designed to integrate common CI platforms directly with standard HPC job schedulers. By operating as a standard unprivileged user and requiring minimal added configuration, hpci makes automated, continuous analysis on HPC highly accessible for computational biologists. We present five use cases to demonstrate its value across diverse biological analyses (single-cell transcriptomics, population genomics, AI-driven structural biology, computational epidemiology) and institutional infrastructures.

## 2 Architecture/system design

hpci is a command-line utility designed to execute directly on a CI runner within an automated workflow. The architecture relies on an intermittent SSH polling mechanism to bridge the CI runner and the HPC environment.

Following the cloning of the source repository to the CI runner, hpci authenticates to the target HPC cluster via SSH. It transfers the specified job-submission script to the cluster and submits it to the local job scheduler (e.g. Slurm, PBS). Upon receiving the job ID from the scheduler, hpci closes the SSH session to prevent network timeouts during long-running biological analyses. It then enters a polling loop: re-establishing an SSH session at defined intervals to query the scheduler for the job’s status, and subsequently closing the connection, and repeating until the job completes.

Once the scheduler reports the job as finished, hpci retrieves the remote job log file, parses the final exit status code, and prints the logs directly into the CI console. Having the job logs printed to the CI logs makes it easier to debug if a job was unsuccessful. Crucially, hpci terminates on the CI runner using the exact same exit code generated by the HPC job. This state synchronisation is vital; it guarantees that a failure on the HPC correctly fails the overarching CI pipeline, providing immediate feedback to the researcher and preventing the execution of downstream CI steps. Appending HPC resource utilisation metrics to these logs allows researchers to easily review and optimise their compute requests directly within the CI web interface.

hpci accepts several command-line arguments, including connection parameters and targeted file paths. Most importantly, it allows users to inject CI-native variables (e.g. branch names, commit hashes, or Git tags) directly into the HPC job’s scheduler arguments and environment variables. This allows the HPC workflow to be explicitly parameterised by its CI context. For example, a submission script can use the Git commit hash to automatically generate unique output directories, preventing file collisions from concurrent workflows and explicitly linking the resulting data back to the exact code version that produced it.

## 3 Implementation

hpci is implemented in Haskell. This language was chosen for three reasons: (1) its strong type system allows for easy refactoring and extensibility (e.g. utilizing Algebraic Data Types and exhaustive pattern matching to safely add support for new HPC schedulers); (2) its powerful parsing libraries are ideal for interpreting scheduler outputs; and, (3) it can compile software to a statically linked binary. By embedding core system dependencies (such as libssh2), the resulting standalone binary is simple to install on CI runners. Pre-compiled x86-64 binaries are distributed via GitHub Release as this matches the architecture of the majority of CI runners. However, the documentation also includes guidance for compiling for macOS on ARM64. To use hpci, the latest release binary can be downloaded from GitHub to a CI runner and made executable. This installation can occur as dynamically as a step within the CI pipeline, or alternatively, hpci can be pre-installed on the runner image.

hpci is developed using rigorous software engineering practices. The codebase has extensive automated tests, including integration tests that are run against Docker containers running Slurm and OpenPBS schedulers.

Finally, hpci operates as a standard unprivileged user via standard SSH protocols, meaning it requires no administrative privileges or custom software installation on the target HPC cluster. hpci deliberately does not attempt to manage cryptographic keys or secrets. Instead, it delegates this responsibility to the established security infrastructure of the host CI platforms. Users inject HPC credentials and environment variables using native CI secret managers (e.g. GitHub Secrets or GitLab CI/CD variables), which automatically redact sensitive data from execution logs. To adhere to the principle of least privilege, we strongly recommend generating dedicated, narrowly scoped SSH keys exclusively for hpci, regularly rotating these keys, utilising ephemeral CI runners, careful review of any code contributions, and configuring CI platforms to only execute workflows on pull requests from approved users.

Documentation on how to use hpci is available at https://github.com/Garvan-Data-Science-Platform/hpci. This includes example CI workflows for GitHub Actions and GitLab CI as well as example job submission scripts for Slurm and PBS schedulers.

## 4 Use cases

Five use cases demonstrate the utility of hpci in a diverse range of bioinformatics/computational biology research and HPC/CI contexts.

### 4.1 Single-cell demultiplexing workflow

Our first use case demonstrates hpci coordinating the automated testing of a single-cell transcriptomics pipeline. The workflow, orchestrated by Snakemake, processes Cell Ranger-generated HTO (Hashtag Oligonucleotide) count matrices to demultiplex cellular barcodes into individual samples using Seurat::HTODemux(). Conda is utilised to provision the required software environments directly on the compute nodes. This CI pipeline leverages Data Version Control (DVC) for validation. When a code change is pushed, hpci triggers the Snakemake workflow on the HPC cluster; subsequently, DVC verifies that the cryptographic hashes of the resulting output files exactly match the cached truthsets, instantly flagging any regressions.

### 4.2 Validation of a variant joint-calling pipeline

Our second use case highlights hpci operating within a national-scale HPC environment utilising the PBSPro job scheduler. Here, a production bioinformatics group employs GitHub Actions to validate a Nextflow-orchestrated genomic joint-calling pipeline. Self-hosted ephemeral runners are used to accommodate lengthy workflow runs and avoid timeouts. Software dependencies are managed via Singularity containers alongside standard HPC environment modules. Crucially, hpci seamlessly passes Git-context environment variables from the CI runner directly into the HPC submission script, enabling dynamic test execution. Standard commits trigger rapid pipeline validation using an NA12878 sample (Zook *et al*. 2014) restricted to chromosome 21. Commits that are tagged to indicate that this version of the code is a candidate for a ‘release’ automatically initiate comprehensive testing on the full dataset. Exact cryptographic reproducibility is unfeasible due to run-specific metadata in file headers and the inherent non-determinism of parallelised joint-calling. These variabilities are primarily found at low quality sites and repetitive genomic regions susceptible to stochastic effects (Regier *et al*. 2018). Instead, the automated workflow uses Illumina’s hap.py to benchmark the output variant calls against a known gold-standard truthset. Minimum performance thresholds for precision and recall are defined in the CI workflow for single-nucleotide polymorphisms (SNP) and insertion or deletion (indel) mutations, ensuring biological accuracy and functional equivalence.

### 4.3 Validation of AI-driven structural annotation

Our third use case demonstrates the utility of hpci in bridging CI with specialised deep learning infrastructure. Baktfold is an emerging microbial pipeline that leverages protein language models (ProstT5, Heinzinger *et al*. 2024) and structural homology search (Foldseek, van Kempen *et al*. 2024) for the rapid annotation of microbial genomes (Bouras *et al*. 2026a). Because predicting protein structural information via language models requires heavy GPU acceleration and significant memory to use large protein language models (ProstT5 has over 1.2B parameters and requires 11GB of VRAM during inference), the test suite cannot execute on standard CI infrastructure. To solve this, GitHub Actions triggers hpci to submit the validation jobs directly to a national-scale HPC cluster running a Slurm scheduler. The workflow executes within a custom built Apptainer/Singularity container to manage the complex deep learning dependencies and specific hardware requirements (such as AMD GPUs), allowing the researchers to automatically verify that updates to the pipeline do not degrade structural token prediction and protein annotation accuracy.

Source code, including hpci CI workflow and HPC job submission script, is available at https://github.com/gbouras13/baktfold. For a related example, see also Phold (Bouras *et al*. 2026b), a bacteriophage annotation pipeline, with publicly accessible source code and hpci configuration files at https://github.com/gbouras13/phold.

### 4.4 Infectious disease modelling

In our fourth use case, hpci connects CI runners in an institutional GitLab instance to that institute’s HPC platform, which uses the Slurm scheduler. This is used to run small-scale simulations of an agent-based infectious disease model on the HPC platform for the purposes of model validation and verifying outputs are reproducible, in the same environment that is used for full-scale simulations. Previously, these small-scale simulations were run on the CI runner itself. However, the available CI runners had different resource constraints and CPU features, which resulted in intermittent CI failures that could only be resolved by inspecting each CI runner and adjusting the installation steps and simulation scripts in the CI script.

### 4.5 Nuclear physics

While hpci was developed to address reproducibility in bioinformatics, it can be applied in other scientific disciplines. Our fifth use case involves the same institutional GitLab instance and HPC platform as the previous use case, in the context of a computational method for solving scattering equations (MCAS) that is used to analyse low energy nucleon–nucleus scattering data (Amos *et al*. 2003). Here, we use hpci to check that the HPC environment modules required to run MCAS are available and can be loaded successfully. This use case was motivated by previous experiences where HPC environment modules were renamed and/or replaced, causing the job scripts to fail.

Source code, including hpci CI workflow and HPC job submission script, is available at https://gitlab.unimelb.edu.au/rgmoss/mcasopt.

## 5 Discussion

We developed an open-source tool to increase the reproducibility of bioinformatics workflows by integrating modern software engineering validation practices (CI) with HPC environments. hpci is designed to be platform-agnostic; it is compatible with common CI platforms (e.g. GitHub Actions, GitLab CI) and schedulers with either Slurm or PBS-like interfaces (OpenPBS, PBSPro). Through five diverse use cases, we have demonstrated its utility in validating a wide range of bioinformatics and computational biology workflows.

Integrating CI with HPC enables automated testing on production hardware, ensuring consistency and reducing the likelihood of errors in production workflow runs. Frequent small validation jobs save both compute resources and researcher time, creating a safety net that empowers teams to confidently refactor code, optimise workflows and collaborate. Furthermore, hpci increases pipeline portability. By being scheduler- and CI-system agnostic, it enables the same analyses to be run across different systems (assuming consistent data and software environments). This simplifies porting workflows between institutions and provides redundancy during HPC maintenance.

By default, hpci relays the HPC job’s exit code back to the CI runner. This provides useful basic workflow validation, immediately catching runtime failures, configuration errors, or missing dependencies. However, as demonstrated in two of our use cases, the value of this integration is magnified when hpci is paired with tools for evaluating workflow outputs. For example, distributed data management tools (e.g. DVC, git-annex, DataLad, Git LFS) provide version control for inputs, and can be used to verify that outputs are strictly reproducible. For analyses where outputs are inherently non-deterministic, domain-specific benchmarking tools (e.g. hap.py for variant calling) allow researchers to evaluate the accuracy of their workflows against known truthsets.

While hpci improves reproducibility at the workflow level via automated testing, it does not intrinsically address the strict reproducibility of the underlying software environment. An exciting future research direction involves integrating CI with GNU Guix- and Nix-backed functional workflow systems (Wurmus *et al*. 2018, Bedő *et al*. 2020; Ravanan—High-reproducibility CWL runner powered by Guix https://github.com/arunisaac/ravanan). Coupling hpci’s automated CI validation with tools like BioNix or Ravanan could provide a paradigm where, on each code commit, the workflow execution logic, software environment, and input data are strictly controlled, ensuring highly reproducible results.

Ultimately, although integrating CI with HPC infrastructure requires a small initial investment of time, the compounding benefits of early error detection and robust workflow reproducibility make hpci a valuable tool for modern computational biology.

## Data Availability

Source code and binaries can be downloaded from https://github.com/Garvan-Data-Science-Platform/hpci. Source code is archived in Software Heritage via Zenodo (https://zenodo.org/records/19587650).
